# Work Productivity in Rheumatoid Arthritis: Relationship with Clinical and Radiological Features

**DOI:** 10.1155/2012/137635

**Published:** 2012-12-19

**Authors:** Rafael Chaparro del Moral, Oscar Luis Rillo, Luciana Casalla, Carolina Bru Morón, Gustavo Citera, José A. Maldonado Cocco, María de los Ángeles Correa, Emilio Buschiazzo, Natalia Tamborenea, Eduardo Mysler, Guillermo Tate, Andrea Baños, Natalia Herscovich

**Affiliations:** ^1^Section of Rheumatology, Hospital General de Agudos “Dr. Enrique Tornú,” Combatientes de Malvinas 3002, C1427ARN Buenos Aires, Argentina; ^2^Instituto de Rehabilitación Psicofísica (I.R.E.P.), C1428DQG Buenos Aires, Argentina; ^3^Organización Médica de Investigación, C1015ABO Buenos Aires, Argentina; ^4^Práctica Médica Privada, Buenos Aires, Argentina

## Abstract

*Objective*. To assess the relationship between work productivity with disease activity, functional capacity, life quality and radiological damage in patients with rheumatoid arthritis (RA). *Methods*. The study included consecutive employed patients with RA (ACR'87), aged over 18. Demographic, disease-related, and work-related variables were determined. The reduction of work productivity was assessed by WPAI-RA. *Results*. 90 patients were evaluated, 71% women. Age average is 50 years old, DAS28 4, and RAQoL 12. Median SENS is 18 and HAQ-A 0.87. Mean absenteeism was of 14%, presenting an average of 6.30 work hours wasted weekly. The reduction in performance at work or assistance was of 38.4% and the waste of productivity was of 45%. Assistance correlated with DAS28 (*r* = 0.446; *P* < 0.001), HAQ-A (*r* = 0.545; *P* < 0.001) and RAQoL (*r* = 0.475; *P* < 0.001). Lower total productivity was noticed in higher levels of activity and functional disability. Patients with SENS > 18 showed lower work productivity than those with SENS < 18 (50 versus 34; *P* = 0.04). In multiple regression analysis, variables associated with reduction of total work productivity were HAQ-A and RAQoL. *Conclusion*. RA patients with higher disease severity showed higher work productivity compromise.

## 1. Introduction 

Rheumatoid arthritis (RA) is a chronical inflammatory disease of unknown etiology that affects mostly patients at a productive age [1]. 

We have noticed that up to 70% of patients with RA will develop work impairment after 10 years of disease evolution and that the most significant increase in work impairment appears in the first year after the diagnoses [2].

Thanks to the progress made in the therapeutic management of the disease, many patients can continue working, though with different levels of work impairment [3]. In patients with RA, work productivity is affected mostly in those severely affected by the disease. However, patients with low disease activity show lower productivity than those who are under remission [4]. 

In 2009, in a descriptive work in which several centers of our country took part, we stated work impairment of 49% in patients with RA [5]. This fact motivated us to investigate the relationship between disease features and work impairment. The objective of the study is to assess the relationship between work productivity and disease activity, functional ability, quality of life, and radiological damage in patients with RA.

## 2. Patients and Methods

### 2.1. Design

During the period between March 2009 and July 2010, an analytical observational and cross-sectional study was done.

### 2.2. Patients

Consecutive RA patients were recruited from a rheumatology hospital in Ciudad Autónoma de Buenos Aires, Argentina. All participants were >18 years old, fulfilled the 1987 American College of Rheumatology (ACR) RA diagnostic criteria [6] and were proficient in the Spanish language. These patients were working in the last week and they accepted to take part of this research under signed informed consent. We excluded patients with other inflammatory arthropathy, fibromyalgia, illiteracy, or cognitive deficiency.

The following demographic features were assessed: age (years old), genre, level of education (years), socioeconomic level (by modified Graffar scale) [7], disease features: evolution time (months), disease activity and its categories by DAS28 [8], functional ability (HAQ A) [9], life quality (RAQoL) [10], functional class (Hochberg “91”) [11], and radiological damage (Simple Erosion Narrowing Score: SENS) [12, 13], and work features: type of employment (according to the Occupational Uniform International Classification of 1988) [14] and the degree of work physical demand by Pujol scale [15].

To assess work productivity the “Work Productivity and Activity Impairment Questionnaire” for rheumatoid arthritis (WPAI-RA) [16] was used. 

We also assessed if patients had showed changes in their work tasks due to RA and classified them into employed, hourly workers, or occasional workers.

Patients completed all questionnaires in the presence of their physician without assistance.

Instruments used in the study are as the follows.

- The DAS28 is an index similar to the original DAS, consisting of a 28 tender joint count (range 0–28), a 28 swollen joint count (range 0–28), ESR, and an optional general health assessment on a visual analogue scale (range 0–100). The DAS28 has a continuous scale ranging from 0 to 9.4, and the level of disease activity can be interpreted as low (DAS28 ≤ 3.2), moderate (3.2 < DAS28 ≤ 5.1), or high (DAS28 > 5.1) [8].
- The HAQ-A is a self-response questionnaire which is used to measure functional status. Subscale scores range from 0 to 3, with higher scores indicating worse functional status [9].
- The RAQoL consists of 30 questions with yes/no response format. Each affirmative answer carries a score of one point. The total score is calculated as the sum of all the affirmative answers. Scores range from 0 to 30, with higher scores indicating poorer QoL [10].
- The Pujol scale classifies physical demand at work in five degrees: (1) sedentary: sitting or occasionally standing, lifting a maximum of 5 kl weight; (2) mild: walking or standing at a significant degree or when it is necessary to sit most of the time using arms and feet to push or pull objects, lifting a maximum of 10 kl weight (3) medium: usually lifting and carrying objects heavier than 12 kl up to 25 kl; (4) heavy: usually lifting and carrying objects heavier than 25 kl up to 50 kl; (5) very heavy: usually lifting and carrying objects heavier than 25 kl and occasionally heavier than 50 kl [15].
- The WPAI-AR consists of six questions: 1 = currently employed; 2 = hours missed due to health problems; 3 = hours missed due to other reasons; 4 = hours actually worked; 5 = degree of health-affected productivity while working (using a 0 to 10 visual analogue scale (VAS)); 6 = degree of health-affected productivity in regular unpaid activities (VAS). The recall period for questions 2 to 6 is of seven days. Four main outcomes can be generated from the WPAI-GH and expressed in percentages by multiplying the following scores by 100: (1) percentage of work time missed due to health problems = Q2/(Q2 + Q4) for those who were currently employed; (2) percentage of impairment while working due to health problems = Q5/10 for those who were currently employed and actually worked in the past seven days; (3) percentage of overall work impairment due to health problems Q2/(Q2 + Q4) + ((1 − Q2/(Q2 + Q4)) × (Q5/10)) for those who were currently employed; (4) percentage of activity impairment due to health problems Q6/10 for all respondents. For those who missed work and did not actually work in the past seven days, the percentage of overall work impairment due to health will be equal to the percentage of work time missed due to health problems. The WPAI-AR was validated in patients with RA [16]. Work productivity is usually divided into two components: absenteeism and presenteeism. The former refers to work leave of absence related to the disease and the other represents work impairment caused by the disease but being present at work [3].

### 2.3. Statistical Analysis

Descriptive statistics were performed to calculate the means, standard deviations, medians, interquartile ranges, frequencies, and percentages.

Correlation between continuous numerical variables has been done by Pearson coefficient (*r*). For the proportional analysis among groups, chi squared test was applied. Comparison among groups of patients has been done by ANOVA with post-hoc analysis and Student's *t*-test with Levene test. Lineal regression analysis has been done taking the percentage of overall productivity loss as dependent variable. A value of *P* ≤ 0.05 was considered significant.

## 3. Results

### 3.1. Population Characteristics

A total of 90 patients with RA were included in the study. Among the 90 patients, the average age was 50 years old and 71% were female. The sample's disease duration was 72 months since their first rheumatology visit. Demographic and disease features are shown in Table 1.

When this research work was being carried out, all included patients were working; therefore, the answer to the first question of the WPAI-AR was affirmative in all cases. 45% of patients were employed, 40% were working by the hour, and 15% were occasionally working.

**Type of Employment**

32 patients were non-qualified sales and services workers (21/32 were working as household help staff). In Table 2, different types of employment have been observed.

**Degree of Work Physical Demand (J. Pujol)**

**Figure 1:**
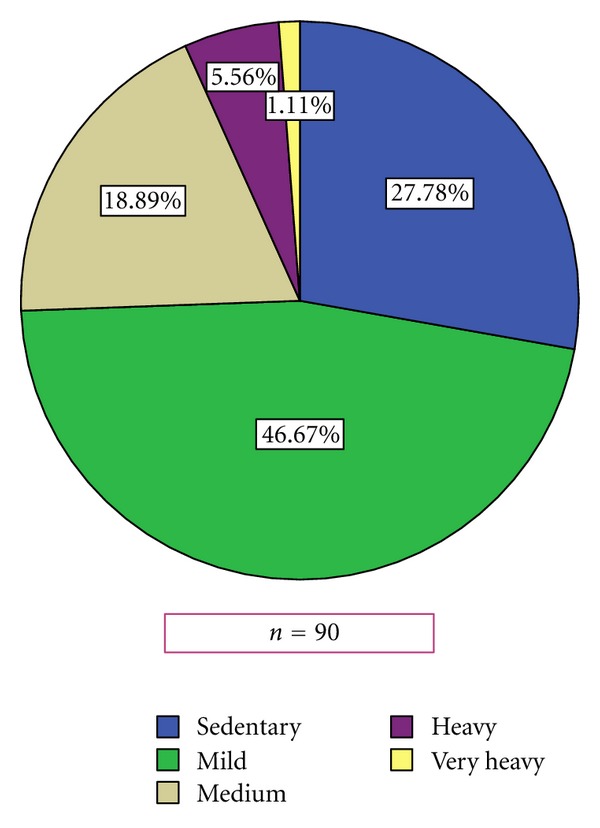
Work physical demand.

Most patients were performing either a mild (46.7%) or sedentary job (27.8%). A minor proportion were doing jobs with intermediate physical demand (18.9%), heavy (5.6%), or very heavy (1.1%) (Figure 1). It is worth mentioning that 65% of patients have modified their tasks due to the disease.

### 3.2. Work Productivity Assessed by WPAI-AR (Table 3)

(1) Absenteeism (missed work hours due to RA): 63% of patients (*n* = 57) did not miss any work hours in the past week (absenteeism = 0%), although 25% of patients miss 8 or more work hours per week. The total average of missed work hours per week was 6.3 (SD 12.6), the average of hours worked during the last week was 34 (SD 20) and the average percentage of presenteeism being of 14%.
(2) Presenteeism(disease impact at work):   88.9% of patients (*n* = 80) presented some degree of work impairment. Among those with and without work impairment, the average percentage of presenteeism or reduction in work performance was 38.4%.
(3) Loss of overall productivity (absenteeism and presenteeism) was 45%.
(4) Impairment of daily life activities (DLA) outside work was 42%.

### 3.3. Correlation of Work Productivity with Disease Activity

Work impairment had a positive correlation with RA activity assessed by DAS28 (*r* = 0.446; *P* < 0.001).

 Assessing the correlation between the loss of overall productivity and different activity categories by DAS28 (mild <3.2, moderate 3.2–5.1, or severe >5.1), we have noticed significant statistical differences among them (Table 4). 

**Figure 2:**
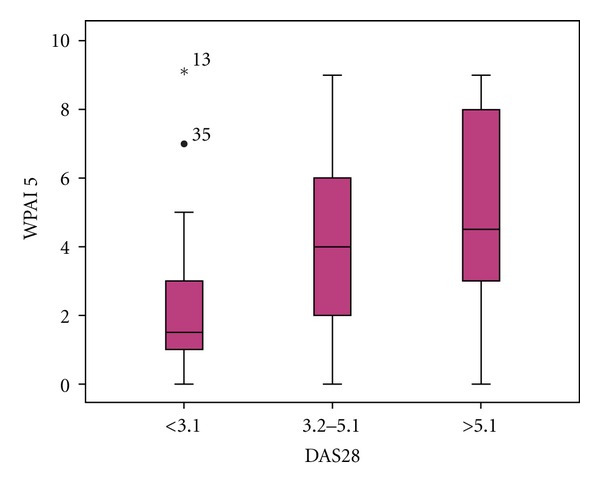
RA disease activity and work impairment.

The degree of work impairment due to RA measured in a numerical scale (0–10) was lower in patients with low disease activity (*P* < 0.01). With the exception of two cases (patient 13 and 35) (Figure 2).

The correlation among lost working hours according to different categories of RA activity by DAS28 (mild, moderate or severe) was assessed and we noticed that 75% of patients with mild RA activity have not shown any loss in work hours, and that only 10% of these lost 6 or more hours a week. However, 50% of patients with severe activity lost at least 8 work hours a week (Table 5).

### 3.4. Correlation between Work Productivity and Functional Ability

Work impairment in patients with severe activity had a positive correlation with functional ability assessed by HAQ A (*r* = 0.545; *P* < 0.001).

The correlation between loss of overall work production and the different levels of HAQ A (<0.5, 0.5 a 0.87 y > 0.87) was assessed. Work impairment was higher (61% IC95: 53–69) in those patients who showed an HAQ-A > 0.87, with significant differences (*P* < 0.01) compared with the other two groups.

Analyzing lost working hours, according to different levels of HAQ A (<0.5, 0.5 a 0.87 y >0.87), we have observed that only 10% of patients with low disability (HAQ A < 0.5) have had a work loss higher than 5 hours. On the other hand, 50% of patients with HAQ A > 0.87 lost no less than 5 working hours a week.

**Figure 3:**
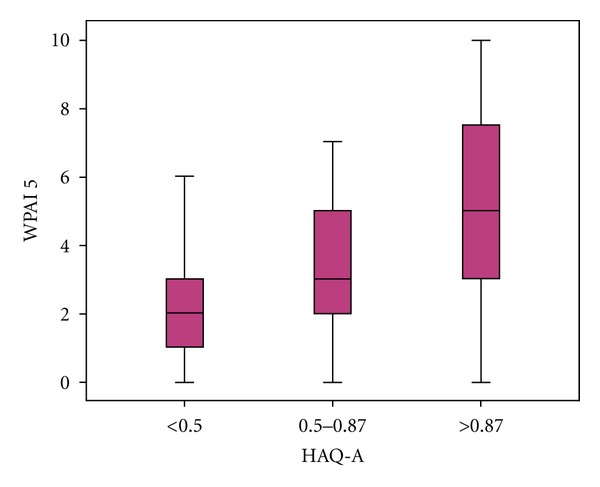
Functional status and work impairment WPAI (range 0–10).

Degree of work impairment due to RA was higher in patients with HAQ A > 0.87 (*P* < 0.01) (Figure 3). 

### 3.5. Correlation between Work Productivity and Life Quality

Impairment of work productivity due to RA had a positive correlation with lower life quality assessed by RAQoL (*r* =  0,475; *P* < 0.001). Patients that showed lower life quality (RAQoL ≥ 6) had a higher work productivity loss (50%) than those with lower values (overall work productivity loss 27%) (*P* < 0.01).

### 3.6. Correlation between Work Productivity and Radiological Damage

Work impairment due to RA had not a significant correlation with radiological damage assessed by SENS (*r* = 0,2*; P* = *NS*).

Dividing patients according to SENS median ≥18 (*n* = 31) versus SENS <18 (*n* = 28), we found a lower loss of overall productivity in those with less radiological damage (50 ± 31 versus 34 ± 25; *P* = 0.04).

### 3.7. Results of Multivariate Analysis

In the multiple regression analysis, considering work impairment as dependent variable, we found the HAQ-A and the RAQoL as unique associated variables. This model had a prediction power of 51% (adjusted *R* ^2^ = 0.51) (Table 6).

## 4. Discussion

In this work, we have found that work impairment in working patients with RA was of 45%. Those patients with higher degrees of disease activity assessed by DAS28 showed higher compromise of work productivity (in absenteeism as well as presenteeism). Our results are consistent with what Zhang and his partners found, who reported a moderate association between disease activity and absenteeism and a strong association with work impairment or presenteeism in 137 employed patients with early RA [17]. 

On the other hand, we have not found any association between disease activity and work productivity in a study done by Geuskens and partners in patients with inflammatory arthropathy of less than 12 moths of evolution [18].

Functional ability, assessed by HAQ, is one of the most frequent predicting factors associated with work impairment in several published studies [2, 19, 20]. We have also described an association between absenteeism and work impairment or presenteeism with functional ability [5]. Patients with RA disability corresponding to HAQ > 1.5 show a significant higher number of missed work days and of days with work impairment ≥50% than those with HAQ <0.5 [21]. Hazes and partners observed that patients with RA treated with certolizumab pegol and methotrexate that achieved a significant clinical improvement as regards pain and physical function reported significant higher increase in work productivity than those who did not achieve the same health improvement [22]. In our study, work impairment in patients with RA showed correlation with functional ability assessed by HAQ-A (*P* < 0.001*), being significantly higher in those patients that showed * HAQ-A > 0.87.

We have found a positive association between work impairment and lower quality of life assessed by RAQoL (*P* < 0.001*), and those patients with poor quality of life *(RAQoL ≥ 6) had more work productivity loss than those with better quality of life (*P* < 0.01).

As regards structural damage, we have not noticed any correlation with work productivity; however, dichotomizing the radiological compromise assessed by SENS according to the median value, we noticed that those patients with more radiological damage showed more work impairment (*P* = 0.04). In previous studies, an association between radiological damage and work impairment or lower indexes of full-time employment [23] has been described [17, 24], but as in our work, radiological compromise had no correlation with work productivity [24].

According to our findings, presenteeism was more compromised than work absenteeism (38.4% versus 14%, resp.). Besides, there was a great number of patients that were not absent at work (with 0% absenteeism), but that did show work impairment due to the disease. This is consistent with what was observed by Zhang and partners [25] who postulate that their results could be due to the fact that other factors would influence work absenteeism besides the disease features.

In our country, work disability figures ranging from 21% to 47% [21–27] have been informed. Studies have shown different factors associated with work disability in patients with RA, such as like a HAQ-A > 0.87, living under poverty line, functional classes III and IV, and a longer evolution of the disease.

Maldonado Ficco and partners informed in a study on 483 patients with early RA that 21% were unemployed, showed higher levels of disease activity, and worse functional ability, and had attended less school years than those who were working [26]. In another multicenter study done in our country over 172 employed patients, 40% of them showed a high risk of work instability (discrepancy between functional abilities of an individual and his/her work tasks). Besides, such instability was associated with HAQ-A ≥ 0.87, presence of erosions and functional class III and IV [28]. We have found that lower functional ability and worse quality of life are factors associated with work impairment

A limitation of this study is that patients with a lot of years of disease evolution could have changed their jobs adapting it to their limitations; in fact 65% of these patients have previously changed their work tasks.

## 5. Conclusion

In this study, we observed that patients with RA that show lower functional ability, lower life quality, higher levels of activity, and bigger radiological damage have a higher number of missed work hours (absenteeism) and higher work impairment (presenteeism). Factors associated with higher work impairment are lower functional ability and worse quality of life. Although at present thanks are to the improvement in the treatment of RA, a lot of patients can continue working. We could observe in this study that those with a bad control of the disease, in spite of being working, show different degrees of work impairment. Therefore, this aspect should be considered when assessing these patients' treatment evolution. 

## Figures and Tables

**Table 1:** Demographic characteristics.

Patients (*n*)	90
Age (mean ± SD)	50 ± 11
Female	64 (71%)
Years of schooling (mean ± SD)	10.2 ± 4.2
Socioeconomic level (*n *= 65)	
I	0
II	3 (4.6%)
III	20 (30.8%)
IV	39 (60%)
V	3 (4.6%)
Months of RA evolution (mean RIQ)	72 (24–120)
DAS 28 (mean ± SD)	4 ± 1
HAQ A (mean, RIQ)	0.87 (0.37–1.5)
RAQoL (mean ± SD)	12 ± 7
Functional class (*n *= 90)	
I	27 (30%)
II	47 (52%)
III	16 (18%)
IV	0
SENS (*n *= 59)	
(mean, RIQ)	18 (11–38)

SD: standard deviation; RIQ: range interquartile.

**Table 2:** Types of employment, according to the occupational uniform international classification.

	*n* (%)
Nonqualified sales and services workers	32 (35.6)
Office employees	14 (15.6)
Shop and market assistants	13 (14.4)
Metallurgy, mechanic construction, and kindred operators	10 (11.1)
Personal service and security service workers	6 (6.6)
Teaching professionals	6 (6.6)
Intellectual and scientific professionals	5 (5.7)
Construction operators	3 (3.3)
Facilities and machines operators and riggers/fitters	1 (1.1)

Total	90 (100)

**Table 3:** Work productivity according to WPAI-AR.

				Percentiles		
	*n *	mean	DS	25	median	75
Missed work hours due to RA	90	6.3	12.6	0	0	8
Missed work hours due to other reasons	90	5.2	13.8	0	0	6
Actually worked hours	90	34	20	18	32	48
Work affected by RA (0 a 10)	90	3.8	2.6	2	3.5	6
DLA impairment due to RA (0 a 10)	90	4.2	2.7	2	4	7.00
Percentage of absenteeism	90	14	24	0	0	20
Percentage of presenteeism	90	38.4	26	20	35	60
Percentage of overall productivity loss	90	45	30	20	45	70
Percentage of DLA compromise	90	42	27	20	40	70

WPAI: Work Productivity and Activity Impairment Questionnaire.

DLA: Daily life activities.

**Table 4:** Loss of overall productivity and RA activity.

DAS28	Percentage of overall work impairment		
	Media	IC 95%	
**<3.2** *n* = 26	**25**	15–36	*P* < 0.01

**3.2–5.1** *n* = 39	**46**	39–56	*P* < 0.01

**>5.1** *n* = 23	**62**	51–74	*P* < 0.01

**Table 5:** Loss of work hours and RA activity.

DAS28	Work hours loss percentiles						
	5	10	25	50	75	90	95
<3.2	0.00	0.00	0.00	0.00	0.00	6.00	9.30
3.2–5.1	0.00	0.00	0.00	0.00	6.00	15.90	36.00
>5.1	0.00	0.00	0.00	8.00	24.00	48.00	60.00

**Table 6:** Multiple lineal regression for work impairment.

	Non standardized coefficients		Standarized coefficients			IC 95% de *B *	
	*B *	Standard error	*β*	*t *	Sig.	Lower limit	Upper limit
(Constant)	10.840	10.470		1.035	0.306	−10.200	31.880
HAQ	21.610	7.568	0.505	2.856	0.006	6.402	36.818
EVA pain	0.111	0.152	0.103	0.731	0.468	−0.195	0.418
DAS28	−1.842	2.948	−0.096	−0.625	0.535	−7.767	4.082
RAQoL	1.094	0.507	0.276	2.156	0.036	0.074	2.113
SENS	0.155	0.213	0.084	0.728	0.470	−0.274	0.584
RA duration	−0.044	0.049	−0.105	−0.907	0.369	−0.142	0.054

Dependent variable: percentage of overall productivity loss.
